# The circadian clock component BMAL1 regulates osteogenesis in osseointegration

**DOI:** 10.3389/fped.2022.1091296

**Published:** 2022-12-21

**Authors:** Shiyong Deng, Meiyao Qi, Ping Gong, Zhen Tan

**Affiliations:** ^1^ State Key Laboratory of Oral Diseases, National Clinical Research Center of Oral Diseases, West China Hospital of Stomatology, Sichuan University, Chengdu, China; ^2^ Department of Implantology, West China Hospital of Stomatology, Sichuan University, Chengdu, China

**Keywords:** implant treatment, Bmal1 gene, osseointegration, osteogenesis, cell senescence, aging

## Abstract

Congenital and developmental craniofacial deformities often cause bone defects, misalignment, and soft tissue asymmetry, which can lead to facial function and morphologic abnormalities, especially among children born with cleft lip and palate. Joint efforts from oral maxillofacial surgery, oral implantology, and cosmetic surgery are often required for diagnosis and treatment. As one of the most widely performed treatment methods, implant-supported cranio-maxillofacial prostheses have been widely applied in the course of treatment. Therefore, stability of peri-implant bone tissue is crucial for the long-term success of treatment and patients’ quality of life. The circadian clock component brain and muscle aryl hydrocarbon receptor nuclear translocator-like protein 1 (BMAL1) was found to be involved in the cell fate of bone marrow mesenchymal stem cells, which were essential in the fixation of titanium implants. This study aimed to investigate the effect of BMAL1 on osteogenesis in osseointegration, providing a brand new solution to increase bone implant conjunction efficiency and implant stability, paving the way for a long-term satisfactory therapy outcome.

## Introduction

Cleft lip and palate (CLP) are one of the most common craniofacial congenital malformations in humans (1). It has been estimated lately that, with ethnic and geographic variation, approximately 1.5–1.7 of every 1,000 infants are born with CLP, which sorely hampers the development of important physiological functions, including breathing, swallowing, speech, chewing, and esthetics (2, 3). At the end stage of growth and development, in order to re-establish esthetics, phonetics, function, and self-confidence, rehabilitation of edentulous space should be the priority (4). Initially reported by Verdi et al., dental implant therapy gradually earned its place in the field of CLP treatment and rehabilitation (5). Severe clinical studies have verified its satisfying results and high long-term success rates (6–8).

Dental implants have been widely employed to restore the function and esthetics for dentition defect or edentulism, especially for youngster patients with congenital craniofacial deformities in an increasing number. The key to a successful dental implant therapy is to achieve direct and complete implant-to-bone contact, which has been defined as osseointegration (9). Following the placement of titanium implants, a series of biochemical reactions occur in the bone marrow, among which osteoprogenitor cells and osteoblasts play a vital role (10). Bone marrow mesenchymal stem cells (BMSCs) are recruited to implant surface and, subsequently, differentiate into osteoblasts. Osteoblasts can secrete bone matrix, directing the process of osseointegration. However, senility leads to changes in the bone marrow microenvironment. At the cellular level, aging impairs the osteogenic differentiation ability of BMSCs and encourages differentiation into adipose cells, leading to fewer osteoblasts and weaker osseointegration (11). Despite the donor's age, an aging bone marrow microenvironment also forces foreign transplanted mesenchymal progenitor cells to transform into adipocytes (12). Therefore, osseointegration in elderly individuals requires a longer period, which remains a clinical challenge. It is essential to study the age-related function of mesenchymal cells, so as to promote a curative effect of dental implants.

Interestingly, several recent studies have shown that the circadian clock system in mammals may influence the function and cell fate of mesenchymal cells (13–15). Brain and muscle aryl hydrocarbon receptor nuclear translocator-like protein 1, briefly BMAL1, is the core component of circadian clock system in mammals, functioning as the positive arm in the negative feedback regulation mechanism of circadian rhythm (16). Bmal1 gene knockout mice show reduced bone volume and lower bone density. Histological examination also demonstrates that deficiency of BMAL1 leads to osteocytes’ decrease *in vivo* (14). The mechanism of aging process has been studied as well. The absence of Bmal1 results in a premature aging process and shortened lifespan in mice (17). Chen et al. found that BMAL1 was related to aging process in aged BMSCs. The expression level of BMAL1 decreased, while the aging of BMSCs hampered osteogenic differentiation (13). Moreover, follow-up studies have demonstrated that the regulation effect of BMAL1 might go through the Wnt pathway (18, 19).

Since BMSCs are the key to osseointegration, we hypothesized that BMAL1 might directly influence osteogenic differentiation of BMSCs among the implant–bone environment. In aged mice, downregulation of Bmal1 gene occurs naturally, impairing the integration between titanium implants and bone. Rescued expression of BAML1 at implant sites can promote local bone healing and obtain higher bone–implant contact ratio (BIC). Hence, in this study, we performed knockdown of BMAL1 of BMSCs to examine its effect on fate choice as well as investigated the overexpression results of lentiviral vector BMAL1 applied around titanium implants in aged mice.

## Materials and methods

### Animals and cell culture

All animal care and studies were approved by the Animal Research Committee of Sichuan University (Chengdu, China) and conducted in accordance with international standards. Young adult (1 month old) and aged (12 months old) C57BL/6 male mice were obtained from the Animal Centre of Sichuan University.

Young adult mice were used for primary extraction of mesenchymal cells. After the mice were sacrificed, the femurs and tibias of both sides were obtained and immediately immersed into phosphate buffer saline (PBS, Gibco) containing 10% penicillin–streptomycin (Liquid, Gibco). The epiphyses were cut off and the marrow cavities were syringed with a sterile medium using a #25-gauge needle. Cells were washed with PBS by centrifugation and then cultured in alpha minimum Eagle's medium (α-MEM, HyClone) with 10% fetal bovine serum (FBS, Gibco) and 1% penicillin–streptomycin. BMSCs were incubated at 37 °C with 5% CO_2_, and the culture medium was exchanged every 2 days. When the confluence rate reached 80%–90%, BMSCs were detached by 0.25% trypsin and then passaged. BMSCs were employed for the subsequent research at passage 4 (18). For osteogenic induction, osteogenic medium was supplemented with the basic medium 10 mM β-glycerophosphate (Sigma), 50 μM ascorbic acid (Sigma), and 10 nM dexamethasone (Sigma).

### BMAL1 knockdown

Bmal1 siRNA and negative control RNA were synthesized from Shanghai Sangon Biotech Co. (China). The sequence of Bmal1 siRNA is GCAAACUACAAGCCAACAU, the bases of which were shuffled for control siRNA. According to the manufacturer's protocol, the siRNA and Lipofectamine® RNAiMAX (Invitrogen) were dissolved in Opti-MEM (Gibco). The two transfection solutions were mixed and then incubated at room temperature in dark for 30 min. When the confluence rate of BMSCs reached 40%–50%, the culture medium was exchanged into α-MEM with 10% FBS without penicillin or streptomycin 2 h in advance. Then, the transfection mixture with Bmal1 siRNA or control siRNA was added into the culture medium. The knockdown efficiency was detected by quantitative reverse transcription polymerase chain reaction (qRT-PCR) after 12-h incubation. If BMAL1 was successfully knockdown, cells were applied for tests as described below.

### CCK-8 assay

A cell counting kit-8 (CCK-8) (Beyotime, China) was used to measure the proliferation ability of BMSCs. At the time point of 1 and 3 days after being transfected with siRNA, cells were cultured into a 96-well plate in five duplicated wells. Then, a 100 µl CCK-8 working solution was added into each well. After incubating for 2 h, absorbance of the culture medium was detected at 450 nm.

### Assessment of senescence-associated β-galactosidase staining

A senescence-associated β-galactosidase (SA-β-gal) staining kit (Beyotime, China) was used to detect cell senescence of BMSCs. Passage 4 BMSCs were incubated in a six-well plate. After being transfected with siRNA, the medium was removed and cells were rinsed with PBS twice. Then, BMSCs were fixed with 4% paraformaldehyde and stained with a working mixture of β-galactosidase and X-Gal at 37 °C overnight in dark. Senescent cells were inspected with an optical microscope and counted in random field of vision.

### RNA extraction and qRT-PCR

In the *in vitro* test, total RNA was isolated using Trizol Reagent (Invitrogen) 7 days after osteogenic induction. The total RNA was treated with DNase and then reverse transcribed *via* a PrimeScript RT reagent Kit (Takara). Real-time PCR was conducted in a 20-µl mixture using SYBR Premix Ex Taq (Takara) and LightCycler 96 (Roche). Bmal1 and osteogenesis-related genes such as Sp7, Runx2, and Bmp2 were examined quantitatively using the housekeeping gene GAPDH as baseline. The primers are shown in Table 1 (18).

**Table 1:** Primer sequences for qRT-PCR.

Primer	Forward	Reverse
Bmal1	5′-AACCTTCCCGCAGCTAACAG-3′	5′-AGTCCTCTTTGGGCCACCTT-3′
Gapdh	5′-ACTGAGGACCAGGTTGTC-3′	5′-TGCTGTAGCCGTATTCATTG-3′
Sp7	5′-TATGCTCCGACCTCCTCAAC-3′	5′-AATAGGATTGGGAAGCAGAAA-3′
Runx2	5′-GGTACTTCGTCAGCATCCTATCAG-3′	5′-GCTTCCGTCAGCGTCAACAC-3′
Alp	5′-AACCCAGACACAAGCATTCC-3′	5′-GCCTTTGAGGTTTTTGGTCA-3′
Dlx5	5′-CTGGCCGCTTTACAGAGAAG-3′	5′-CTGGTGACTGTGGCGAGTTA-3′
Col1a1	5′-TAGGCCATTGTGTATGCAGC-3′	5′-ACATGTTCAGCTTTGTGGACC-3′
Bglap	5′-TTGGTGCACACCTAGCAGAC-3′	5′-ACCTTATTGCCCTCCTGCTT-3′

qRT-PCR, quantitative reverse transcription polymerase chain reaction.

### Alkaline phosphatase staining and protein assay

After 7 days of osteogenic induction, BMSCs were fixed with 4% paraformaldehyde and then incubated with a working mixture of BCIP/NBT (BCIP/NBT Alkaline Phosphatase Color Development Kit, Beyotime, China) for 15 min. Images were taken with the Epson Perfection V370 Photo Scanner. For the alkaline phosphatase (ALP) protein assay, the total protein of BMSCs was collected with a protein extraction kit (PE001, Sab-biotech) and quantitatively assayed using the BCA Protein Assay Kit (Beyotime, China). An alkaline phosphatase assay kit (Beyotime, China) was used for the protein assay. After reaction with 0.5 mM p-nitrophenyl phosphate for 10 min, absorbance of p-nitrophenol formed by hydrolysis was detected at 405 nm. The corresponding of ALP activity was converted according to the standard curve.

### Alizarin Red S staining

After 14 days of osteogenic induction, BMSCs were fixed with 4% paraformaldehyde and stained with Alizarin Red S (ARS) solution (Solarbio, China). Images were taken with the Epson Perfection V370 Photo Scanner.

### Implant surgery

The mouse Bmal1 sequence (NM_007489) was retrieved from GeneBank, and the lentiviral vector was obtained from GENECHEM Co. (China). Mice were divided into three groups: (1) young group: young adult mice without lentiviral vector injection; (2) aged group: aged adult mice without lentiviral vector injection; and (3) transfection group: aged adult mice with lentiviral vector injection. Rod-shaped titanium implants (commercial pure titanium, grade 4, SLA surface modification, 2 mm in length and 1 mm in diameter) were obtained from WEGO CO. (China). Mice were anesthetized through intraperitoneal injections of ketamine (70 mg/kg) and xylazine (10 mg/kg), and implants were inserted into the distal aspect of femurs as described by Xiang et al. (20) and Xue et al. (21). Briefly, after dissecting the skin and muscles, the implant sites were prepared by sequential drilling with 0.8/1.0 mm-diameter stainless-steel twist drills under continuous sterile saline irrigation. Then, one implant was press-fitted into the prepared hole (Figure 3A). In the transfection group, BMAL1 lentiviral vector was injected into the holes before implant insertion. Finally, the muscles and skin were sutured tightly with 5-0 nylon suture (Huawei, China) to protect the implant sites from the surrounding environment. Figures 3B,C show the representative actual and x-ray inspection of femurs with implants.

### Identification of lentiviral vector transfection

To confirm the functionality of BMAL1 lentiviral vector transfection system in the peri-implant sites, the IVIS Spectrum imaging system (PerkinElmer, Inc.) (absorbance of 465 nm) and immunofluorescence labeling of GFP (Abcam) were used to observe the expression of GFP in the transfection group at day 14 after the implant surgery.

### Micro-CT analyses

Two weeks after implant surgery, femurs containing implants were harvested and scanned with a µCT 80 micro-CT system (SCANCO 50, Switzerland) at 8 µm resolution (90 kV, 200 µA, 500 ms integration time). A radius of µ20 m around implants was defined as the peri-implant site for volume of interest (VOI). After three-dimensional reconstruction, a few basic parameters were analyzed as follows: (1) BIC: bone–implant contact; (2) BV/TV: bone volume fraction; (3) Tb. N: trabecular number; (4) Tb. Sp: trabecular separation.

### Histomorphometric analyses

The specimens were fixed with 4% paraformaldehyde, dehydrated with a graded series of ethanol solutions (60%, 80%, 90%, and 100%), and embedded in light-curing epoxy resin (Technovit 7200VLC, Hereaus Kulzer, Germany) for 1 week. Then, the specimens were sliced perpendicular to the long axis of the implants and sanding to about 50 µm thickness using a grinding system (Exakt Apparatebau, Germany). Sections were stained with Stevenel’s blue and Van Gieson’s Picrofuchsin stain (20), and images were taken under a light microscope (OLYMPUS BX43F, Japan). For histomorphometric analysis, BIC and BV/TV were measured using the NIH IMAGE J software (21). BIC was defined as the line percentage of the implant interface that directly contacts the bone. As for the BV/TV, it indicated the proportion of bone to total tissue volume around the implants.

### Statistical analyses

All data were presented as mean ± standard error (SD). Statistical differences were calculated *via* Student's *t* test for independent samples or one-way ANOVA for multiple comparisons using SPSS 17.0 software (SPSS, Inc., Chicago, IL, United States). A *P* value of less than 0.05 was considered as a statistically significant difference.

## Results

### The knockdown of BMAL1 accelerated cell senescence of BMSCs

**Figure 1:**
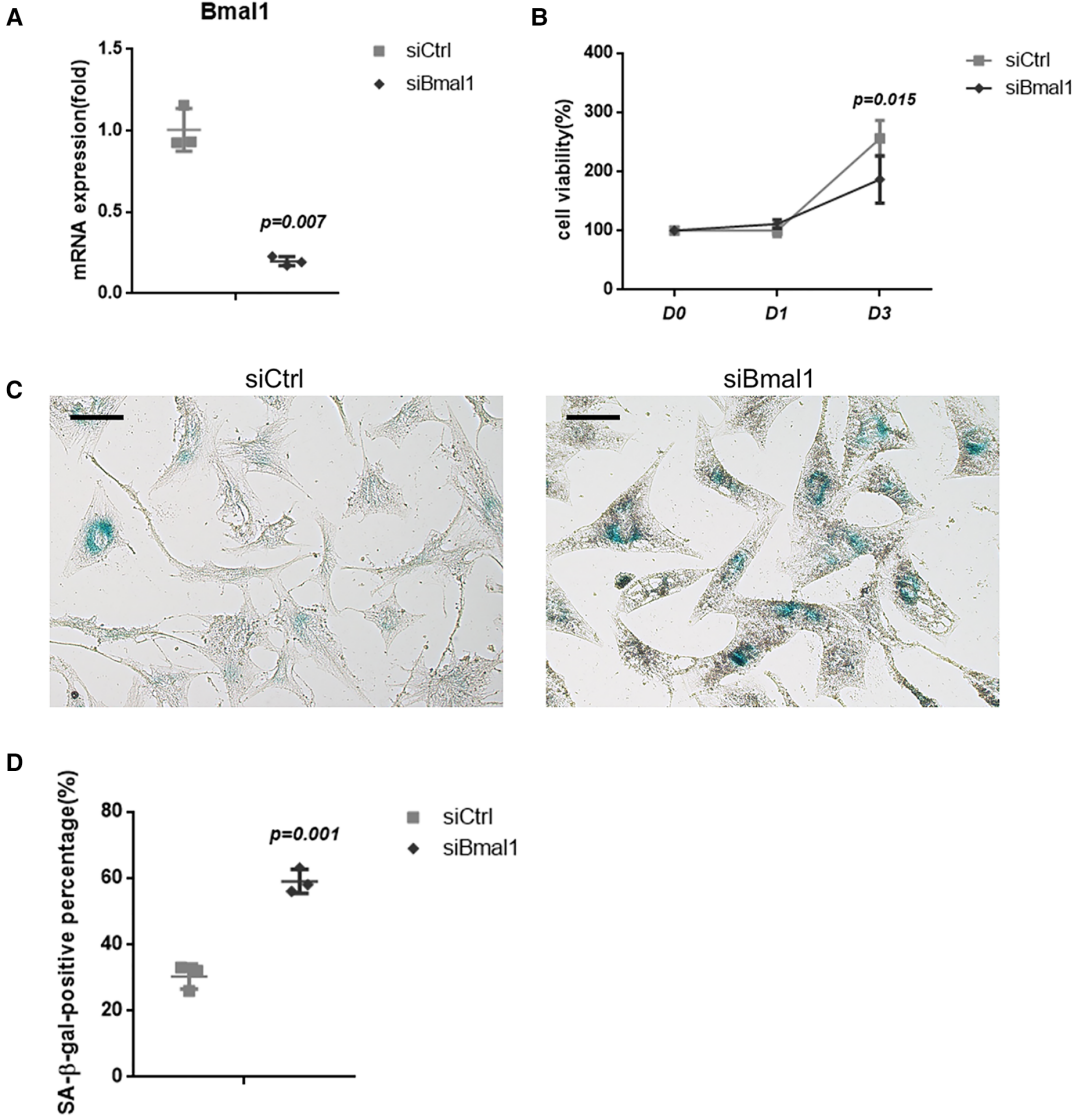
Depletion of BMAL1 accelerated cell senescence of BMSCs. (**A**) qRT-PCR test verified the knockdown efficiency of siBmal1. (**B**) Cell viability of BMSCs after siRNA treatment. (**C**) SA-β-gal staining images of BMSCs after siRNA treatment. Scale bar = 100 µm. (**D**) Quantitative analyses of SA-β-gal staining. BMSCs, bone marrow mesenchymal stem cells; qRT-PCR, quantitative reverse transcription polymerase chain reaction; SA-β-gal, senescence-associated β-galactosidase.

BMAL1 was knockdown in BMSCs to verify its effect on cell senescence. qRT-PCR tests showed the efficiency of BMAL1 knockdown (Figure 1A). CCK-8 assay demonstrated that deficiency of BMAL1 led to reduced cell proliferation capacity (Figure 1B). In the knockdown group, more blue-dyed products catalyzed by β-galactosidase were observed than another group under optical microscope *via* SA-β-gal staining (Figure 1C). It indicated that the knockdown of BMAL1 in BMSCs accelerated cell senescence. The counting result of senescent cells is shown in Figure 1D.

### The knockdown of BMAL1 reduced osteogenic differentiation of BMSCs

**Figure 2:**
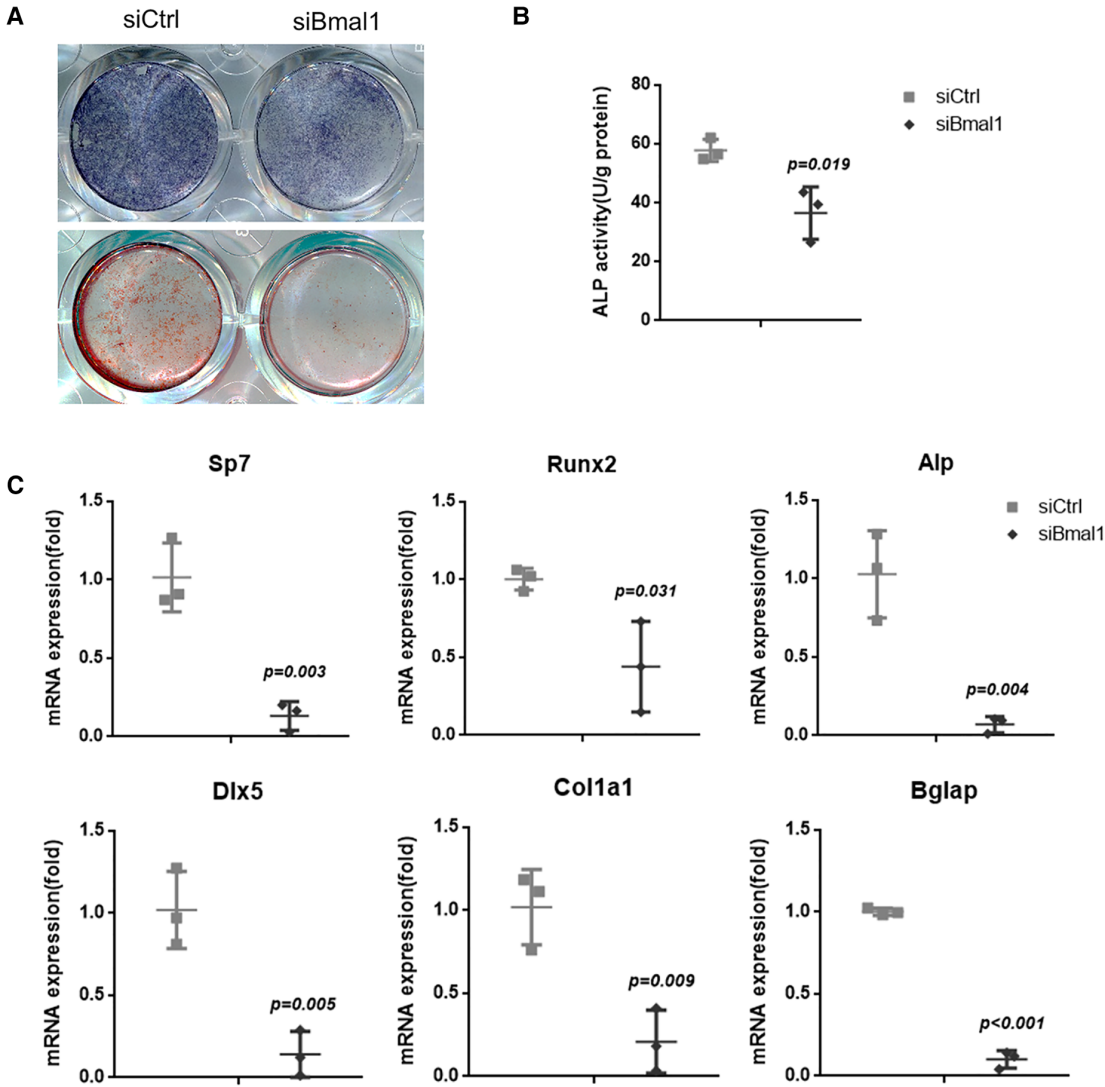
Knockdown of BMAL1 reduced osteogenic differentiation of BMSCs. (**A**) Representative images of ALP and ARS staining of BMSCs in siBmal1 and siCtrl group. (**B**) Quantitative analyses of ALP activity. (**C**) qRT-PCR analyses of the expression of osteogenesis-related genes (Sp7, Runx2, Alp, Dlx-5, Col1a1, and Bglap) 7 days after osteoinduction. BMSCs, bone marrow mesenchymal stem cells; qRT-PCR, quantitative reverse transcription polymerase chain reaction; ALP, alkaline phosphatase; ARS, Alizarin Red S.

The osteogenesis of BMSCs was examined with ALP staining after 7 days osteoinduction, which indicates reduced capacity of osteogenic differentiation in the knockdown group (Figure 2A). The quantitative analysis of ALP activity also showed the downregulated osteogenesis under BMAL1 deficiency (Figure 2B), and the ARS staining demonstrated the consistent result (Figure 2A). Moreover, qRT-PCR tests at 7 days after osteoinduction revealed that a few osteogenesis-related genes (Sp7, Runx2, Alp, Dlx-5, Col1a1, and Bglap) were downregulated significantly (Figure 2C). The results above indicated reduced osteogenic differentiation of BMSCs under BMAL1 depletion.

### Transfection of BMAL1 promoted titanium implants osseointegration

**Figure 3:**
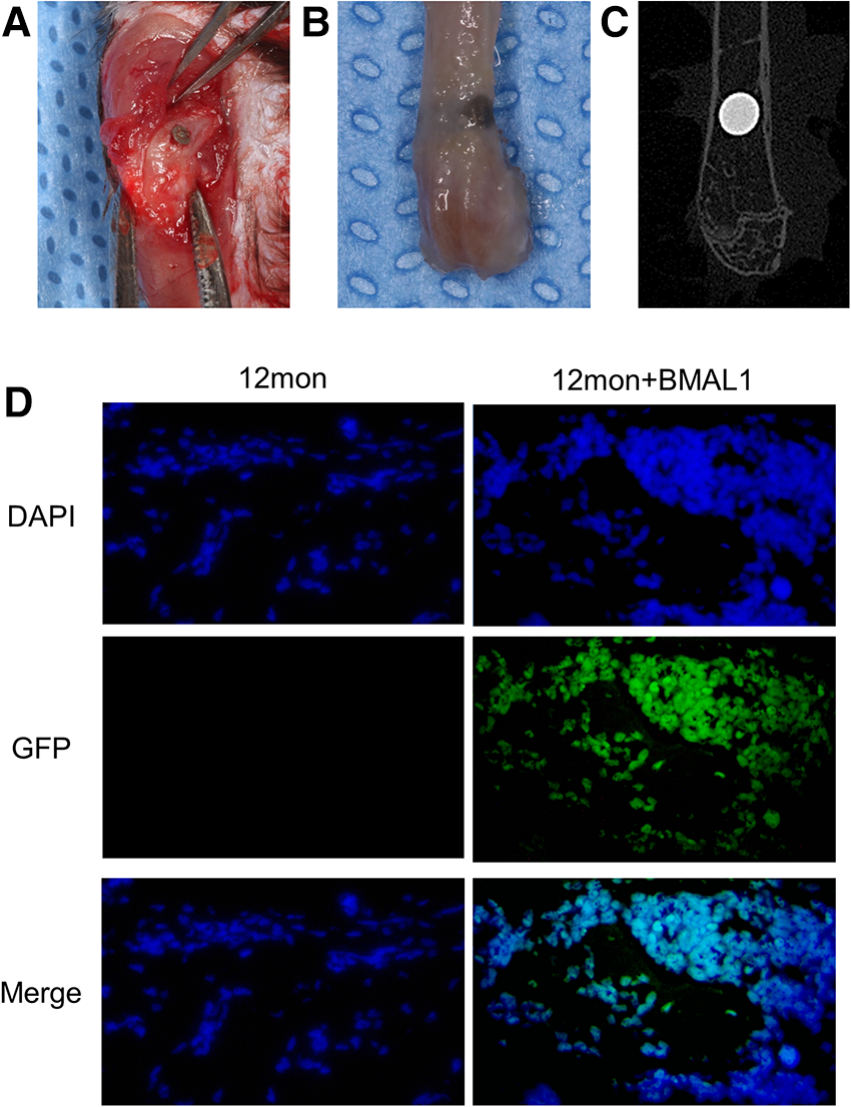
Representative images of implant surgery. (**A**) Titanium implants inserted into the distal end of femurs. (**B**) Representative image of femur with implant. (**C**) X-ray inspection of femur with implant. (**D**) Immunofluorescence proved positive expression of GFP in transfection group.

**Figure 4:**
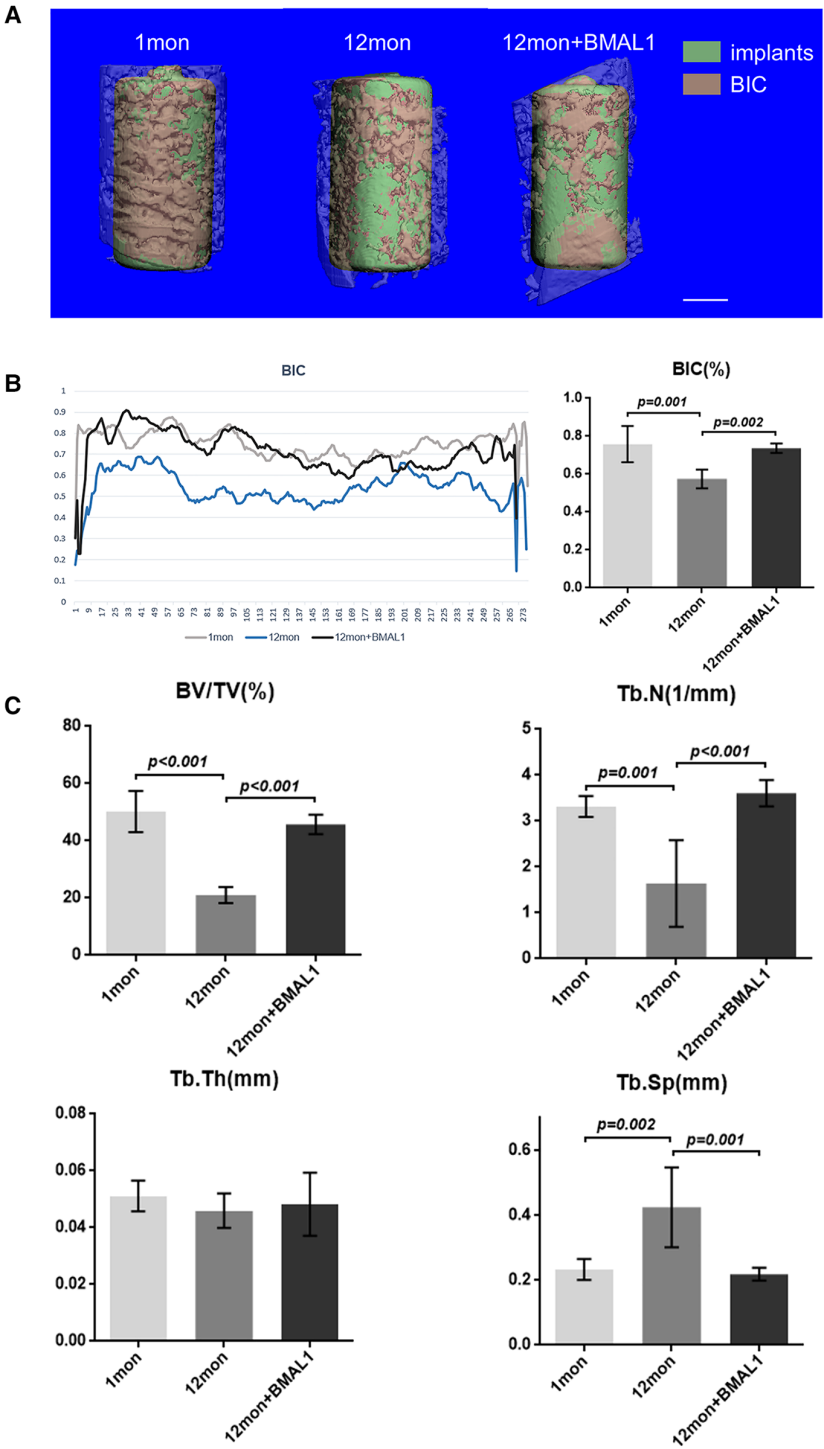
Micro-CT analyses of BIC and trabecular bone of femurs. (**A**) Representative 3D reconstruction images of peri-implant sites. Scale bar = 500 µm. (**B**) Representative liner graph and quantitative analyses of BIC rate. (*n* = 4) (**C**) Quantitative analyses of bone morphology parameters regarding BV/TV (%), Tb. N (1/mm), Tb. Th (mm), and Tb. Sp (mm). (*n* = 4). BIC, bone-to-implant contact; BV/TV, bone volume fraction; Tb. N, trabecular number; Tb. Sp, trabecular separation.

**Figure 5:**
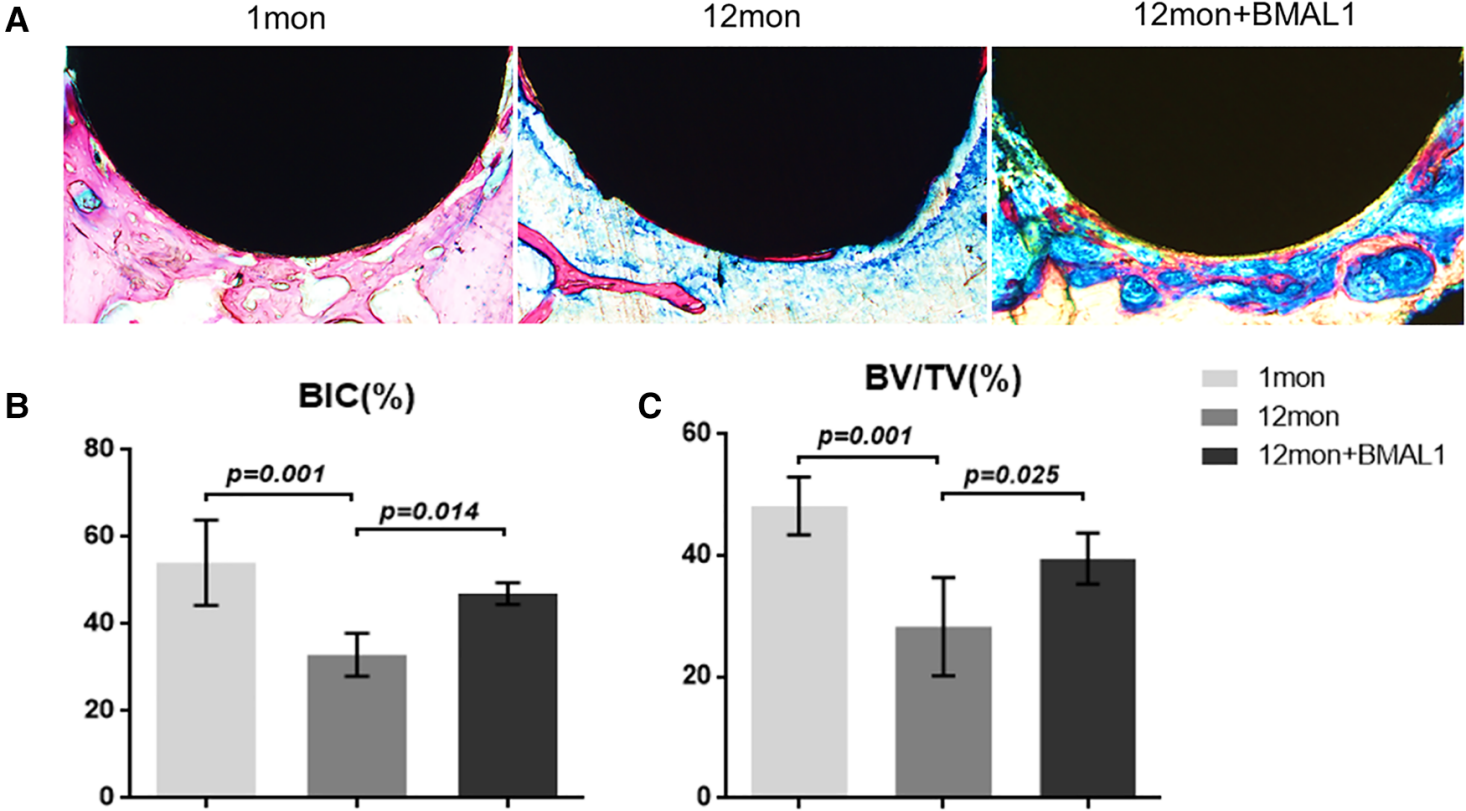
Histologic analyses of femurs with implants. (**A**) Representative image of histological sections after 2 weeks healing. (**B**,**C**) Quantitative analyses of BIC (%) and BV/TC (%), respectively, through histological images. (*n* = 4). BIC, bone-to-implant contact; BV/TV, bone volume fraction.

To investigate the overexpression of BMAL1 around titanium implants in aged mice, micro-CT and histomorphometric analyses were conducted. First of all, immunofluorescence proved that GFP is expressed around the implant sites in the transfection group (Figure 3D). The three-dimensional reconstruction of the implant and newly formed bone tissue indicated that 2 weeks after implant surgery, bone–implant contact was obviously detected in the young group, while in the aged group, less bone–implant contact could be observed. After overexpressing BMAL1, samples in the transfection group exhibited increased BIC (Figure 4A). Histomorphometric analyses demonstrated likewise that the transfection of BMAL1 reversed bone–implant contact, which decreased while aging (Figure 5A). Figures 4B and 5B show the calculation results of BIC. As shown in Figure 4C, the BV/TV and Tb. N of mice femurs around implants in the aged group were significantly lower than that in the young group, but they increased in the transfection group. Otherwise, the Tb. Sp of mice femurs around implants in the aged group were significantly higher than that in the young group, but they decreased in the transfection group. Histomorphometric analyses demonstrated consistent results of BV/TV (Figure 5C). Results above indicated that overexpression of BMAL1 facilitates osseointegration, which decreases while aging.

## Discussion

Implant treatment has become a mainstream of dental rehabilitation therapy in recent years. Despite the relatively high successful rate of implant therapy and a great number of surface modification techniques for titanium implants, osseointegration in certain individuals, such as the elderly, remains a challenge (22, 23). After the placement of implants, osteoprogenitor cells around the sites are recruited, differentiating into osteoblasts following the hemostasis and inflammatory phase (10). Osteoblasts derived from mesenchymal stem cells secrete extracellular bone matrix on the surface of implants and mineralize the matrix. Aging status, however, alters this process (11). In recent years, increasing amount of evidence shows that disruption of biorhythm can lead to various pathologies in different tissues (17, 24, 25). Simultaneously, interruption of circadian clock causes aging, clicking with the fact that BMAL1 has been reported as the key factor to aging, in addition to its effect on circadian rhythm in mammals (17, 26).

The effects of circadian clock proteins on bone metabolism and osteogenesis have been extensively studied, while the conclusion remains controversial though. Chen et al. found that gene and protein levels of BMAL1 in BMSCs decreased during the aging process, suggesting the potential impact of BMAL1 in osteogenesis, and it was confirmed by subsequent studies (13, 18). Related studies have been carried out in mice as well. Mice deficient of Bmal1 show low bone mass phenotype, meanwhile increasing bone resorption (14, 27). It has been reported that bone resorption can also be regulated by BMAL1 (28). However, another report has reached a contradicted conclusion (15). More detailed research on bone mass regulation should be carried on in the near future as a result. Interestingly, Zhuo et al. demonstrated that BMAL1 along with PER2, another component circadian clock protein, might play a synergistically negative role in bone metabolism. Qian et al. also reported this opposite effect (29, 30). Though a whole set of genes, including BMAL1, Per2, and Npas2, all have been reported to add up to the basic biorhythm regulation, BMAL1 can still be regarded as one of most important genes and studied most, according to earlier investigations, which is the reason we chose to lay our eyes on BMAL1 in this study. Same as the results of Chen et al., we herein demonstrated that knockdown of BMAL1 in mouse BMSCs decreased the osteogenic differentiation ability (Figure 2). In addition, the results in this study confirmed that deficiency of BMAL1 also influenced the cell aging status of BMSCs (Figure 1). These results suggested that BMAL1 might have an influence on bone metabolism around dental implants due to its key function of BMSCs in osseointegration under a common microenvironment.

As for osseointegration, few studies have focused on the function of circadian clock proteins in dental implants so far. Mengatto et al. conducted whole genome microarray analyses to evaluate the total RNA around implants in rats and provided evidence that the circadian rhythm system was involved in the establishment of osseointegration (31). The mentioned circadian clock genes, including Bmal1, Per2, and Npas2, act together as the positive and negative arms of the negative feedback mechanism. NPAS2 was proven to facilitate osseointegration of titanium implants through a neuroskeletal mechanism (32). However, as far as we know, this study is the first on the effect of BMAL1 in osseointegration directly. During this study, our results confirmed that BMAL1 regulated osteogenesis around titanium implants. The ability of bone healing of mammals decreased in the aging process with deficiency of BMAL1 function (11, 13). Thus, the bone-to-implant contact was impaired in aged mice and the overexpression of BMAL1 reversed the osteogenesis ability to facilitate the fixation of implants.

Although we have found that the circadian clock component BMAL1 indeed facilitates osseointegration through a mouse model, there are several limitations. First of all, transfection efficiency of BMAL1 vector should be precisely calculated after applied in BMSCs of femur. Moreover, femurs arise from mesoderm embryologically, while alveolar bones where dental implants are placed in reality are from neural crest cells. Differences in the developmental origins lead to discrepancy in bone healing (33). Due to the difficulty in lentiviral vector injection in alveolar bones, implant models are merely conducted in femurs. Otherwise, osseointegration is regulated by a complex molecular network related to bone quality, the transient chondrogenic phase, the vitamin D axis, and the circadian rhythm (34). The molecular mechanism and whether the circadian clock component can influence the other aspects of osseointegration, such as angiogenesis, remain uncertain. As He et al. once proved that Wnt/β-catenin pathway was involved in the regulation of BMAL1 in bone marrow stromal cells (18), it should be considered in further studies. In this study, we use lentiviral vector to accomplish sustained BMAL1 overexpression around implants as it has been considered an effective transgene expression approach (35), while gene knockout mice can achieve more stable gene scavenging models. Thus, further studies with improved models may contribute to a deeper understanding about the effect of BMAL1 on titanium implants osseointegration.

To conclude, this study revealed that the circadian clock component BMAL1 might regulate aging-related impaired osteogenesis around titanium implants *in vivo*, which may be a result of reversing the senescence of BMSCs, and facilitate their osteogenesis ability. The BIC and relevant bone mass parameters are rescued through lentivirus transfection of BMAL1. The regulatory molecule network of osseointegration remains an attractive topic and the circadian clock system may play an essential role. Our results provide a specific sight of peri-implant osteogenesis regulation and further studies are needed.

## Data Availability

The original contributions presented in the study are included in the article/Supplementary Material, further inquiries can be directed to the corresponding author.
